# Caregiving Self-efficacy and Knowledge Regarding Patient Positioning Among Malaysian Caregivers of Stroke Patients

**DOI:** 10.7759/cureus.7390

**Published:** 2020-03-24

**Authors:** Chai-Eng Tan, May-Yin Hi, Nur Sarah Azmi, Nur Khairina Ishak, Fathin alyaa Mohd Farid, Aznida Firzah Abdul Aziz

**Affiliations:** 1 Department of Family Medicine, Faculty of Medicine, Universiti Kebangsaan Malaysia, Kuala Lumpur, MYS

**Keywords:** stroke, caregiver, self-efficacy, knowledge, caregiver education, home care, nursing

## Abstract

Background

Most family caregivers of stroke patients in Malaysia do not receive adequate prior preparation or training. This study aimed to determine levels of patient positioning knowledge and caregiving self-efficacy among caregivers of stroke patients.

Methods

This cross-sectional study was conducted at an urban teaching hospital involving 128 caregivers of stroke patients. The caregivers were conveniently sampled and completed the data collection forms, which comprised their socio-demographic data, patients’ functional status, the Caregiving Knowledge For Stroke Questionnaire: Patient Positioning (CKQ-My© Patient Positioning) to measure caregiver’s knowledge on patient positioning, and the Family Caregiver Activation Tool (FCAT©) to measure caregivers’ self-efficacy in managing the patient. Descriptive and multivariate inferential statistics were used for data analysis.

Results

Among the caregivers sampled, 87.3% had poor knowledge of positioning (mean score 14.9 ± 4.32). The mean score for FCAT was 49.7 ± 6.0 from a scale of 10 to 60. There was no significant association between knowledge on positioning and self-efficacy. Multiple linear regression showed that caregivers’ age (B = 0.146, p = 0.003) and caregiver training (B = 3.302, p = 0.007) were independently associated with caregivers’ self-efficacy.

Conclusion

Caregivers’ knowledge on the positioning of stroke patients was poor, despite a fairly good level of self-efficacy. Older caregivers and receiving caregiver training were independently associated with better caregiver self-efficacy. This supports the provision of caregiver training to improve caregiver self-efficacy.

## Introduction

When a stroke survivor develops long-term disability, the role of caregivers is of utmost importance in the rehabilitation and nursing care of the patient. The majority of stroke caregivers in Malaysia are informal caregivers, comprising family members of the patients [1-2]. Very few Malaysian families can afford paid caregivers, whether trained or untrained [1]. Family caregivers shoulder the responsibilities of providing direct care, emotional support, coordinating care, financial support, and advocacy for the patient [3].

Caregivers need basic caregiving knowledge and skills to care for stroke patients in view of the various disabilities and potential complications of the stroke. These include performing or aiding the patients in basic activities of daily living such as feeding, mobilizing, toileting, bathing, and dressing. They also require higher-level skills such as supervision and administration of medications, handling medical equipment (e.g. dressing, regular turning), managing the healthcare system (appointments, refilling prescriptions), arranging transportation, and being the patient’s advocate [4]. 

The prevalence of caregiver burden ranged from 25% to 54% worldwide [5]. Informal caregivers have been found to have a higher prevalence of depression, as well as the negative effects of their physical, psychological, and social health [6]. In Malaysia, stroke caregivers also face unmet needs in terms of information regarding problem-solving and medical-related knowledge, financial, and social support [2]. The burden faced by caregivers has been associated with various negative impact toward patients’ well-being, such as increased post-stroke depression, cognitive decline, physical disability, and general quality of life [7-9]. 

Caregiver training may reduce the burden and improve caregivers' quality of life and confidence in providing care to the patient [10-12]. The provision of information alone has been shown to improve caregivers’ knowledge and satisfaction [13]. In various developed countries, transition care programs have been developed to meet the need for caregiver training and support [14-15]. During the transition process, structured caregiver training and post-discharge support and follow-up are provided to ensure patient and caregiver safety. The implementation of transition care is highly variable as they are tailored to the available social and community health services at respective locations. 

In Malaysia, most health facilities have yet to implement structured caregiver training for caregivers of stroke patients [16]. There are limited studies regarding the unmet needs of caregivers of stroke patients. A study conducted among informal caregivers in the Klang Valley showed that 78% wanted to have more information to solve their problems, as well as 67% who wanted more information and advice from medical specialists [2]. This suggests most Malaysian informal caregivers have low caregiving knowledge. Caregivers need basic caregiving knowledge such as patient positioning, feeding, patient transfer, and pressure ulcer prevention to prevent complications or adverse events both to patients and caregivers alike [17]. Caregiver training may also help improve the self-efficacy of caregivers, reduce anxiety in providing care, prevent caregiver burnout, and promote better outcomes and quality of life for the stroke patient [10,18]. 

To date, there are no local studies that measure knowledge specific to caregiving for stroke patients. Hence, this study aims to determine the level of caregiving knowledge among caregivers and their level of confidence in caring for stroke patients.

## Materials and methods

This cross-sectional study was conducted at an urban teaching hospital from February 2017 to October 2017. The study population involved adult caregivers (aged 18 years and above) of stroke patients who had been diagnosed for more than 3 months and attending rehabilitation activities at the department. Both primary and secondary caregivers were eligible for study inclusion. The respondents were conveniently sampled in the waiting area of the rehabilitation department. Only Malaysian caregivers who were able to understand English or Malay were recruited. Paid formal caregivers and family caregivers for the institutionalized patients were excluded from the study. 

The sample size was calculated to determine the population mean, using Yamane’s formula for a population of 188 patients who attend rehabilitation sessions over 6 months with an error tolerance of 0.05 [19]. A minimum sample size of 128 patients was required for this study. 

Data was collected using an interviewer-assisted questionnaire consisting of 4 sections: 1) sociodemographic data of the caregiver; 2) caregiving profile; and 3) Caregiving Knowledge Questionnaire (CKQ-My©: Patient Positioning) and Family Caregiving Activation Transitions (FCAT). Interviewers assisted in reading out the items and filling in their responses. 

Study tools

The CKQ-My© was a validated bilingual questionnaire that measured caregiving knowledge with 2 subscales: positioning and feeding [20]. The positioning subscale (CKQMy-Positioning) had 28 items where respondents were required to indicate which pictures of patient positioning were correct. The positioning subscale showed good internal consistency reliability (Cronbach alpha 0.70). The cut-off score for good knowledge was arbitrarily set at 20 for positioning subscale, based on the mean score from the pilot study [20]. This cut-off score was applied because the pilot study was conducted on a population similar to the current study. The feeding subscale was not used for this analysis in view that it was too easy for most caregivers.

The FCAT was used to determine the caregivers’ level of self-efficacy for performing several generic tasks related to the care of a patient with chronic diseases [21]. These tasks included managing patient medications, appointments, care plan, and awareness of where to obtain relevant medical information. The possible range of scores for the FCAT was from 6 to 60. The 10-item FCAT was translated and underwent face validation and pilot testing. The translated FCAT had good internal consistency reliability (Cronbach alpha 0.78) from the pilot study [20]. Higher FCAT scores demonstrated better self-efficacy in caring for the patients. There is no cut-off score to categorize respondents with good or poor self-efficacy in caregiving.

Data analysis

Data were analyzed using IBM SPSS Statistics Version 25. Descriptive data were described using frequencies, percentages, means, standard deviations, medians, and interquartile ranges. Multiple linear regression was used to determine the association between the FCAT scores and the caregivers' sociodemographic and caregiving characteristics. All independent variables with clinical significance were entered into the model for multiple linear regression. The independent variables were checked to ensure normality of residuals and absence of collinearity, to fulfill assumptions required to conduct multiple linear regression. Independent variables that were found to violate the assumptions were examined and removed from the model where appropriate. Alpha was set at 0.05 for statistical significance.

Ethical approval

This study received ethical approval from the UKMMC Medical Research and Ethics Committee (FF-2017-196). Permission to translate and adapt the CKQ-My and FCAT were obtained from the original developers of the questionnaire. Caregivers received verbal and printed brief information about the study and provided written consent prior to their participation. All data were kept confidential and only accessible to the research team members.

## Results

A total of 128 caregivers were included in this study. The mean age of our study sample was 45.8 ± 16.81 years. Most caregivers were female (64.8%, n = 83), Malay (64.8%, n = 83), married (66.4%, n = 85), had up to secondary level education (62.5%, n = 80) and represented the middle income group (50.8%, n = 65). Most of them (60.2%, n = 77) were primary caregivers and majority (75.8%, n = 97) reported that they did not receive any training. More than half of the caregivers surveyed were caring for severely dependent patients, with a modified Rankin score (MRS) of 4-5 (n = 69, 54%). About 72.6% (n = 94) of caregivers surveyed had been caring for patients who had been diagnosed with stroke for less than 5 years. Table 1 displays the characteristics of our respondents. 

**Table 1:** Background characteristics of respondents

Background characteristics	n (%)
Gender	
Male	45 (35.2)
Female	83 (64.8)
Race	
Malay	83 (64.8)
Chinese	34 (26.6)
Others	11 (8.6)
Marital status	
Single	36 (28.1)
Married	85 (66.4)
Divorced/Widowed	7 (5.5)
Education	
Primary level	14 (11)
Secondary level	80 (62.5)
Tertiary level	34 (26.6)
Household income	
< RM1000	34 (26.6)
RM1000 – RM4999	65 (50.8)
>RM5000	29 (22.6)
Role	
Primary caregiver	77 (60.2)
Secondary caregiver	51 (39.8)
Duration of diagnosis	
0 – 5 years	94 (72.6)
6 – 10 years	24 (18.8)
>10 years	11 (8.6)
Modified Rankin Score (MRS)	
Independent to moderately dependent (MRS 1 – 3)	59 (46)
Severely dependent to totally dependent (MRS 4 – 5)	69 (54)
Received caregiver training	
Yes	31 (24.2)
No	97 (75.8)
Information source	
Doctors	50 (39.1)
Nurses	27 (21.1)
Internet or other informal source	24 (18.8)
Other hospital personnel	23 (18)
Other caregivers	10 (7.8)
Books or other formal sources	4 (3.1)
Non-governmental organisations	2 (1.6)
Others	8 (6.3)

The mean knowledge score for patient positioning was 14.5± 4.22 (Table 2). The majority of caregivers (85.9%) had poor knowledge of positioning. 

**Table 2:** Knowledge scores for patient positioning sd, standard deviation; IQR, interquartile range

Knowledge for positioning (Range of scores 0-29)	n (%)
Poor knowledge (<20)	110(85.9)
Good knowledge (>20)	18 (14.1)
Mean score (sd)	14.5 (± 4.22)
Median score (IQR)	14 (11, 18)

The mean score for FCAT was 49.7(±6.0) and shown in Table 3. While there are no local population norms to define the cut off scores for high or low self-efficacy, the mean FCAT score appeared to be at the higher range of scores, suggesting good self-efficacy. Positioning knowledge was not correlated with the caregivers’ self-efficacy (r = 0.046, p = 0.607).

**Table 3:** Caregiving self-efficacy scores FCAT, Family Caregiver Activation in Transitions; IQR, interquartile range; sd, standard deviation

FCAT Score	Mean (sd)	Median (IQR)
Total FCAT Score (Range of scores 10-60)	49.7 (6.0)	50 (46, 53.8)
I am able to make sure he/she goes to every scheduled medical appointment.	5.51(0.70)	6 (1)
I know when, how much, and how his/her medications should be taken.	5.26 (0.92)	6 (1)
If he/she needs help from a healthcare professional, I am confident I can insist until I get what is needed.	5.24 (0.79)	5 (1)
I maintain an accurate list of his/her medications.	5.23 (0.96)	5 (1)
I have or will check with the doctor to make sure which medications he/she should be taking (including how often and how much)	5.16 (1.02)	5 (1)
I understand which tasks in his/her care plan that should be prioritized.	4.95 (0.93)	5 (2)
I make sure a written list of questions is taken along with him/her to every medical appointments.	4.68 (1.06)	5 (1)
I have a trusted healthcare personnel whom I can contact if I have questions about medications.	4.63 (1.26)	5 (2)
I know the signs that show his/her condition is getting worse and how to respond.	4.54 (1.15)	5 (1)
I keep a written record of his/her health conditions, allergies, medications, along with the names and phone numbers of treating health professionals.	4.49 (1.22)	5 (2)

Multiple linear regression analysis was done to identify independent associations between the FCAT scores with independent variables such as caregivers’ age, gender, race, education, income, caregiver status, caregiver training, duration of care, knowledge score in positioning and patients’ modified Rankin score (Table 4).

**Table 4:** Multiple linear regression for independent associations with FCAT scores * p<0.05; FCAT, family caregiver activation tool; MRS, Modified Rankin Score

Independent variables	Unstandardized coefficient, B	Standard error	Standardized coefficient, β	t	p
Constant	41.181	3.376		12.198	<0.001
Age	0.146	0.48	0.406	3.06	0.003*
Married	-0.863	1.189	-0.080	-0.726	0.469
Secondary education	-0.014	1.378	-0.001	-0.010	0.992
Tertiary education	-1.136	1.532	-0.084	-0.741	0.460
Middle income	2.272	1.387	0.151	1.639	0.104
High income	-0.353	2.962	-0.010	-0.119	0.905
Primary caregiver	-0.682	1.284	-0.055	-0.531	0.596
Received caregiver training	3.302	1.205	0.236	2.741	0.007*
Patient’s MRS	-0.164	0.431	-0.033	-0.380	0.704
Duration of caregiving	0.075	0.120	0.055	0.623	0.535
Position knowledge score	0.222	0.129	0.154	1.719	0.088

The regression equation was significant (F = 2.777 (11, 114), p = 0.003), with R2 of 0.211. The caregiver’s predicted FCAT score was 41.18 + 0.146(Age) -0.863 (Married) -0.014 (Secondary education) -1.136 (tertiary education) +2.27 (middle income) -0.353 (high income) - 0.682 (primary caregiver) + 3.302(Caregiver training) -0.162 (Modified Rankin Score) + 0.075 (duration of caregiving) + 0.222 (Positioning knowledge). Only age and caregiver training had a significant linear relationship with the FCAT score. The FCAT score increased by 0.146 for every year of age and by 3.32 with caregiver training.

## Discussion

The findings of this study highlighted the deficiencies of informal stroke caregivers’ knowledge about patient positioning. This appeared to contrast with their perceived self-efficacy in caring for stroke patients. The descriptive analyses also show differences in their self-efficacy for various caregiving tasks.

Stroke patients, particularly those with hemiparesis, are at increased risk of developing complications such as pressure injuries and contractures. Correct positioning after a stroke is an essential component of rehabilitation therapy, as it can help to prevent these complications [22]. Correct positioning can also promote optimal recovery by modulating the muscle tone, ensure stability and enhance comfort [22]. Unfortunately, with the large amount of information related to the stroke itself, caregivers might not have been educated regarding the importance of proper positioning methods. Again, patient positioning is not routinely taught in Malaysian hospitals before the patient is discharged home. To date, there are no published papers reporting on the discharge planning process for stroke patients in Malaysia. The Malaysian clinical practice guidelines on the management of ischemic stroke do not include recommendations on caregiver training [23]. It only includes “assessment of patient’s suitability for rehabilitation” as one of the clinical audit indicators for stroke services.

As the CKQ-My© is the first validated tool in Malaysia to measure stroke caregivers’ knowledge of positioning, there are no equivalent studies for direct comparison. A study among formal caregivers in Korea found that 64.3% of responses regarding positioning were correct [24]. This is a stark contrast from the 85.9% of caregivers with poor knowledge (CKQ-My score of <20) in this study. While the Korean study did not use a validated scoring scale, this still suggests possible differences for knowledge between their population and our study population. However, the Korean caregivers were those who had received training and formally employed to care for stroke patients in various healthcare institutions in Korea. Awareness regarding the importance of proper positioning for stroke patients was also suboptimal among healthcare professionals in Singapore [25]. This suggests that healthcare professionals need to recognize the importance of proper positioning for stroke patients in order to train caregivers before the patients are discharged. 

The FCAT scores represent the self-efficacy of stroke caregivers, where higher scores indicate better self-efficacy. However, the FCAT items do not ask about direct caregiving tasks such as patient positioning. Instead, it asks about their ability to supervise and execute the care plans for the patient, including appointments, medications, and consultations with healthcare professionals. The caregiver’s age and having caregiver training were significantly associated with better self-efficacy. However, the effect of age on the self-efficacy was small. A similar effect of age on caregivers of patients in dementia has been observed [26]. Younger caregivers were at higher risk for burnout [12]. 

The provision of caregiver training helped to improve the self-efficacy of stroke caregivers, which is supported by various caregiver training interventions [10,27]. This is important as better caregiver self-efficacy has been shown to improve stroke patient’s recovery and mental health status [28-29]. Better caregiver self-efficacy also helps to mediate caregiver burden, improve perceived social support and mental well-being [30]. Hence, a structured caregiver training programme in Malaysia is strongly needed to provide support for caregivers. The contents of caregiver training should include knowledge on patient positioning, understanding the patient’s care plan, managing the patient’s health records and knowing what red flag signs to watch out for. 

This study is one of the few studies on caregivers of stroke patients in Malaysia. It gives insight into the knowledge and self-efficacy of caregivers in managing stroke patients at home. The findings provide some insight as to possible content for developing caregiver training programmes. The instrument for measuring caregiver knowledge had content, face, and construct validity [20]. The FCAT had content, linguistic and face validity, as well as good internal consistency reliability. Using multiple linear regression analysis provided a way to statistically adjust for potential confounders to determine the independent associations between FCAT scores with each independent variable. 

However, it is limited by the lack of generalizability to the Malaysian population due to convenience sampling and only included caregivers of stroke patients at one single urban teaching hospital. Hence some selectional bias may be present and the results should be interpreted with caution. The results may not be extrapolated to caregivers residing in other regions of Malaysia.

## Conclusions

In conclusion, caregivers of stroke patients in this study had poor knowledge of patient positioning. The self-efficacy of caregivers were influenced by their age as well as provision of caregiver training. We recommend more studies on the unmet needs for caregivers of stroke patients to be conducted locally. The findings will then be translated into development of caregiver training programmes and policy in ensuring that it is conducted throughout hospitals in Malaysia.

## Figures and Tables

**Figure 1:**
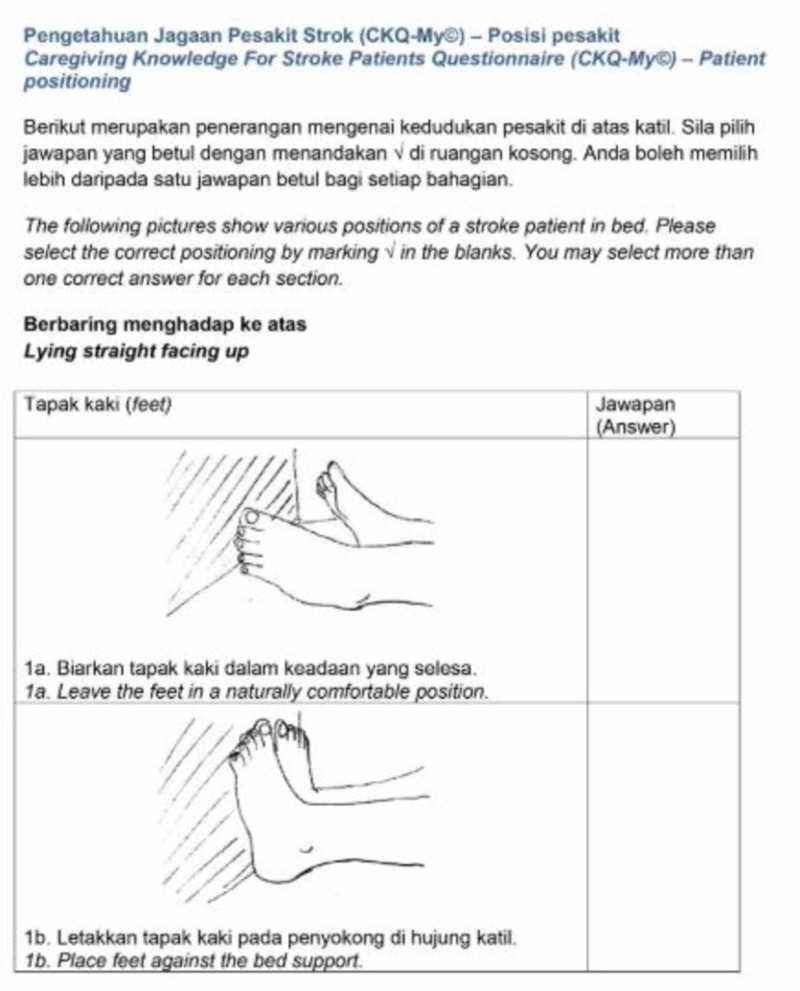
Sample page for CKQ-My Positioning For permissions, please contact Dr Chai-Eng Tan at .my
